# Secondary structure encodes a cooperative tertiary folding funnel in the *Azoarcus* ribozyme

**DOI:** 10.1093/nar/gkv1055

**Published:** 2015-10-19

**Authors:** Anthony M. Mustoe, Hashim M. Al-Hashimi, Charles L. Brooks

**Affiliations:** 1 Department of Biophysics, University of Michigan, Ann Arbor, MI 48109, USA; 2 Department of Biochemistry and Chemistry, Duke University School of Medicine, Durham, NC 27710, USA; 3 Department of Chemistry, University of Michigan, Ann Arbor, MI 48109, USA

## Abstract

A requirement for specific RNA folding is that the free-energy landscape discriminate against non-native folds. While tertiary interactions are critical for stabilizing the native fold, they are relatively non-specific, suggesting additional mechanisms contribute to tertiary folding specificity. In this study, we use coarse-grained molecular dynamics simulations to explore how secondary structure shapes the tertiary free-energy landscape of the *Azoarcus* ribozyme. We show that steric and connectivity constraints posed by secondary structure strongly limit the accessible conformational space of the ribozyme, and that these so-called topological constraints in turn pose strong free-energy penalties on forming different tertiary contacts. Notably, native A-minor and base-triple interactions form with low conformational free energy, while non-native tetraloop/tetraloop–receptor interactions are penalized by high conformational free energies. Topological constraints also give rise to strong cooperativity between distal tertiary interactions, quantitatively matching prior experimental measurements. The specificity of the folding landscape is further enhanced as tertiary contacts place additional constraints on the conformational space, progressively funneling the molecule to the native state. These results indicate that secondary structure assists the ribozyme in navigating the otherwise rugged tertiary folding landscape, and further emphasize topological constraints as a key force in RNA folding.

## INTRODUCTION

RNA molecules perform remarkably diverse cellular functions, many of which require RNAs to fold to specific tertiary structures that rival the complexity of proteins (1). However, unlike proteins that are comprised of a diverse alphabet of 20 amino acids, RNAs are made up of only four chemically similar nucleotides. This lack of chemical diversity poses a fundamental challenge to RNA folding, resulting in a rugged free energy landscape and making it difficult to discriminate against non-native folds (2–4).

The RNA free energy landscape is strongly hierarchical, with RNA folding generally divisible into distinct secondary and tertiary folding steps (5,6). The thermodynamic rules underlying secondary structure folding into double helices are well established (7). However, much less is understood about the thermodynamics governing tertiary folding. Tertiary structure is stabilized by a relatively small number of long-range tertiary interaction motifs, which must offset the large electrostatic and conformational entropy penalties to folding (8,9). Furthermore, many tertiary interactions such as A-minor and base-triple motifs are largely non-specific (10–13), and modular tertiary motifs like tetraloop/tetraloop-receptors (TL/TLRs) are used multiple times in the same molecule (14), seemingly presenting a significant challenge to specific folding. Nevertheless, many RNAs fold rapidly with remarkable specificity (15–18), implying a relatively smooth free energy landscape.

Two proposed and potentially related sources of tertiary folding specificity and stability are the RNA secondary structure scaffold and cooperativity between tertiary interactions. While RNA folding does not always occur in a strictly hierarchical fashion (19,20), native secondary structure plays a vital role in shaping the overall tertiary free energy landscape. In particular, secondary structure imposes steric and connectivity constraints (together termed topological constraints) that greatly limit the conformational space accessible to RNA helices (21–23). Studies of tRNA and small model systems have shown that these constraints impose large free-energy penalties on adopting non-native 3D conformations, hence providing an important source of folding specificity (23–26). Separately, cooperativity between distinct tertiary interactions has been shown to play a critical role in stabilizing the native fold of many RNAs (20,27–29). While incompletely understood, it has been proposed that this cooperativity emerges from topological constraints that couple the conformation of distal helices, reducing the entropic cost of concurrent tertiary interaction formation (25,27).

To date, studies of topological constraints have been limited to relatively small RNAs on the order of 75 nucleotides. However, several lines of evidence suggest a general role for topological constraints in the folding of larger RNAs. Notably, recognizing that secondary structure greatly constrains the number of feasible tertiary folds, Michel and Westhof (30) were able to derive a remarkably accurate three-dimensional model of the group I intron structure. Subsequent studies have since identified persistent correlations between secondary structure and 3D conformation in other large RNAs (31–37).

In this study, we use coarse-grained simulations of the *Azoarcus* ribozyme to establish a novel quantitative model for the role of topological constraints in the tertiary folding of a large RNA. Our coarse-grained model, TOPRNA, isolates topological constraints from other molecular forces to directly interrogate the free-energy contributions of secondary structure in tertiary folding, and has been experimentally validated in several RNA systems (24,26). Consistent with prior studies of smaller RNAs, we find that topological constraints strongly limit the ensemble of conformations accessible to the ribozyme. Unique to large RNAs, these constraints extend over multiple junctions to couple the conformation of distal helices, helping to discriminate against non-native tertiary interactions, and provide the basis of strong tertiary cooperativity critical to the stability of the native fold.

## MATERIALS AND METHODS

### Simulation details

As described elsewhere (24), TOPRNA is a three-bead coarse-grained model that isolates the effects of topological constraints on RNA 3D conformation. Secondary structure base pairs are permanently bonded together and constrained to adopt A-form helical structure, with all other nucleotides treated as freely rotatable chains. Electrostatics and non-bonded attractive interactions are ignored, with the exception of a small attraction exclusively experienced between paired base beads.

Simulations were performed using the updated TOPRNA2 force field (26). Temperature replica exchange was performed using eight exponentially spaced temperature windows spanning 300–400 K using the REPD module of CHARMM (38), with exchanges attempted every 5000 dynamics steps. Exchange rates varied between 0.32 and 0.36. Unrestrained simulations were performed for a total of 5 × 10^9^ dynamics steps, recording conformations at each exchange interval to yield a total of 10^6^ conformations. Other simulation parameters were set as previously described (24).

**Figure 1.**
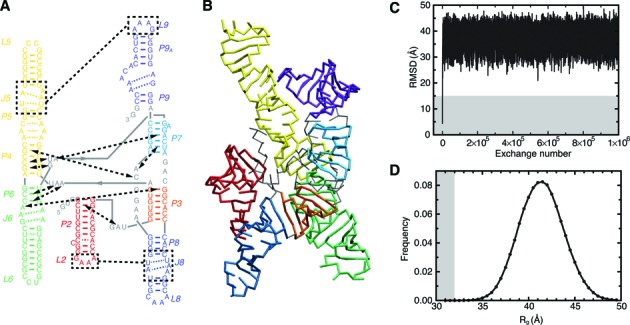
Simulation overview. (**A**) Naming scheme and secondary structure of the *Azoarcus* ribozyme. Solid colored lines indicate canonical base pairs, dashed colored lines indicate noncanonical internal loop base pairs, and dashed black lines indicate important tertiary interactions. (**B**) TOPRNA representation of the native tertiary structure (PDB 1U6B (39)), colored according to (A). (**C**) Root mean square deviation (RMSD) of the TOPRNA simulation from the native structure as a function of replica exchange step. RMSD was computed using all P beads. (**D**) Histogram of the radius of gyration of conformations sampled by the simulation. Gray background indicates native-like RMSD and *R*_g_ values in (C) and (D).

Initial coordinates were obtained from chain B of PDB 1U6B (39) using the –fromc option of the *toprnaCreate.pl* utility (brooks.chem.lsa.umich.edu). The artificial U1A site of the crystal structure was manually deleted from the input PDB and replaced in the TOPRNA model with the natural tetraloop using the default rebuild-from-sequence functionality. Base pairs drawn as solid lines in the Figure 1A secondary structure were enforced as standard TOPRNA base pairs. Non-canonical internal loop pairs inferred from the crystal structure were also enforced (dashed lines in Figure 1A) based on prior work showing that internal loop pairs place important constraints on the RNA conformational space (21,40). These non-canonical pairs were implemented as described in the TOPRNA documentation (brooks.chem.lsa.umich.edu). Briefly, an additional bead was added to one of the bases to fill the appropriate base pair steric volume. The base (B) beads of both residues were then modified to feel a weak short-range attractive interaction to other paired B beads, and both residues were given backbone dihedral potentials with force constants one-fourth the strength experienced by canonically paired residues. Finally, NOE restraints were placed between paired B beads with *r*_min_ = 5.5 Å and *r*_max_ = 7.5 Å, and between S beads of the same residues with *r*_min_ = 11 Å and *r*_max_ = 14 Å, with *f*_max_ = *k*_min_ = *k*_max_ = 2 kcal/mol/Å^2^. Note that unpaired nucleotides inside internal loops remained freely rotatable. Two additional simulations repeated using alternative sets of internal loop restraints exhibited negligible differences (Supplementary Information). Thus, while the microscopic arrangement of internal loop pairs does in theory affect topological constraints (24,40), these effects are finer than the resolution of our current analysis.

Triple-helix (TH) and L9/J5 restrained simulations were performed for a total of 10^9^ steps using identical parameters as above. The TH was enforced by restraining the two backbone dihedrals separating P4 and P6 to their crystal structure values with *K* = 50 kcal/mol, and placing NOE restraints between the S-beads of A39 to C87 and G113; A40 to C86 and G114; G116 to C43 and G83; and U117 to G44 and C82. The L9/J5 interaction was enforced using NOE restraints between the S-beads of A181 to U53 and A72; and A183 to C52 and G74. All NOE restraints were parameterized with *f*_max_ = *k*_min_ = *k*_max_ = 2 kcal/mol/Å^2^, and *r*_min_ and *r*_max_ set to ±1 Å of the values measured in the crystal structure.

Simulations of the separate tetraloop/tetraloop-receptor (TL/TLR) model system were performed for a total of 5 × 10^8^ steps using identical simulation parameters, yielding an exchange rate of 0.54 between replicas. Initial coordinates were generated using the –fromc option of *toprnaCreate.pl*. Residues 26–50 (the hairpin containing the TLR) were initiated from coordinates of the *Azoarcus* ribozyme P5 hairpin. These residues were harmonically restrained, and then remaining residues built using the default assembly procedure. During production simulations, the TLR was restrained using backbone dihedral restraints centered around the P5 crystal structure conformation with *K* = 50 kcal/mol.

### Simulation analysis

Analysis was restricted to conformations sampled at 300 K, discarding the first 10^7^ dynamics steps as equilibration. Interhelical Euler angles (*α*_h_, *β*_h_, *γ*_h_), were measured as previously described (25,41), using 10° bins to compute the fraction of conformations sampled by pairs of helices. For the three-way and four-way pseudoknotted junctions, 30° and 60° bin sizes were used to compute the fraction of unique 2 × (*α*_h_, *β*_h_, *γ*_h_) and 3 × (*α*_h_, *β*_h_, *γ*_h_) conformations sampled, respectively (25). 30° and 60° bin sizes yield ∼10^6^ unique bins (*N*_bin_), comparable in size to the conformational pool (*N*_conf_) generated by our simulations, and providing the best estimate of the constraints on the junction given the sampling limitations of our simulations. However, the use of different bin sizes complicates direct comparisons between the fractions of conformations sampled (*F*_samp_). To facilitate comparisons between the pseudoknots and to tRNA, we thus also compute the normalized fraction, *F*_norm_ = F*_samp_/*F*_trna_. *F*_trna_ is the fraction of conformational space sampled by tRNA (25), and F*_samp_ is computed with 60° bins, subsampling the *Azoarcus* ribozyme conformational pool to keep *N*_conf_/*N*_bin_ constant for each junction. For these calculations, *N*_conf_ = 499 000 was used for the four-way pseudoknot (identical to the *N*_conf_ for tRNA (25)), and *N*_conf_ = 4,621 for the three-way pseudoknot.

Mutual information (MI) between pairs of helices was computed as previously described using 45° bin sizes (25). In order to normalize for the varied entropies of the different (*α*_h_, *β*_h_, *γ*_h_) distributions, we report the normalized mutual information MI_norm_(*X*,*Y*) = MI(*X,Y*)/*H*(*X,Y*), where *H*(*X,Y*) is the Shannon entropy of the joint distribution of the two sets of angles *X* and *Y*. MI_norm_ varies from 0 (no correlation) to 1 (completely correlated).

Δ*G*_topo_ values for forming contacts between residues *i* and *j* were computed from the probability that the distance *d_ij_* between sugar beads *S_i_* and *S_j_* is < 14 Å:

(1) \begin{equation*} \Delta G_{{\rm topo}} = - RT\,\rm {In}\left( {\frac{{P(d_{ij} < 14{\mathring{\rm A}})}}{{1 - P(d_{ij} < 14{\mathring{\rm A}})}}} \right), \end{equation*}

where *R* is the ideal gas constant and *T* is 300 K. The 14 Å cutoff roughly corresponds to the distance across a canonical base pair. Δ*G*_topo_ values for forming a TL/TLR interaction were computed similarly from the probability that *d_ij_* < 14 Å for at least two sets of *i* ∈ TL and *j* ∈ TLR. More restrictive analyses, such as requiring at least four *d_ij_* < 14 Å contacts, increased Δ*G*_topo_ but did not significantly affect the ΔΔ*G*_topo_ between different TL/TLRs. The Δ*G*_topo_ of the J6/P3 interaction was computed using the probability that *d_ij_* < 14 Å for at least two sets of *i* ∈ J6 and *j* ∈ P3. The presence of P4/P6 stacking, used as a proxy of TH-folding, was evaluated using previously described criteria (25,42).

## RESULTS

### TOPRNA simulations sample an extended unfolded ensemble of the *Azoarcus* ribozyme

To explore significance of topological constraints in the folding of large RNAs, we used TOPRNA to simulate the conformational ensemble accessible to the native secondary structure of the 195 nt *Azoarcus* group I ribozyme (Figure 1A and B). This thermostable ribozyme has been well characterized experimentally and is an important model system for understanding large RNA folding (18). Using 5 × 10^9^ steps of temperature replica exchange molecular dynamics simulations, we were able to confidently map the topological constraints on the ribozyme and measure conformational free energy differences >6.5 kcal/mol between different 3D conformations. Convergence was confirmed by comparisons to two additional, independent simulations (Supplementary Information).

As expected of TOPRNA simulations, the ribozyme quickly equilibrated to a heterogeneous ensemble of extended conformations (Figure 1C and B). The ensemble average radius of gyration (*R*_g_) is 42 Å, in reasonable agreement with the experimentally determined radius of hydration (*R*_h_) of 40 Å when secondary structure is folded but tertiary structure is unstable (43), and *R*_g_ = 37 Å when the ribozyme tertiary structure is destabilized via mutagenesis (27). The mean *R*_g_ = 42 Å is also consistent with prior simulations of the NAST coarse-grained model (43). The expansion we observe relative to the crystal structure (*R*_g_ = 30 Å) indicates that topological constraints impose a large entropic cost on adopting compact conformations. Nevertheless, *R*_g_ = 42 Å is substantially more compact than the *R*_g_ > 65–75 Å state observed at low ionic conditions when secondary structure is incompletely folded (27,44), indicating that native secondary structure significantly constrains the ribozyme. Although *R*_h_ and *R*_g_ are coarse descriptors of structure, these observations suggest that despite many simplifications, TOPRNA yields a reasonable approximation of the secondary structure ensemble absent strong tertiary interactions. As proposed previously (24), this may be because attractive interactions are negated by residual electostatic repulsion at low-to-moderate solution ion concentrations, causing conformational entropy to dominate ensemble behavior.

### Topological constraints significantly limit *Azoarcus* ribozyme conformation

**Figure 2.**
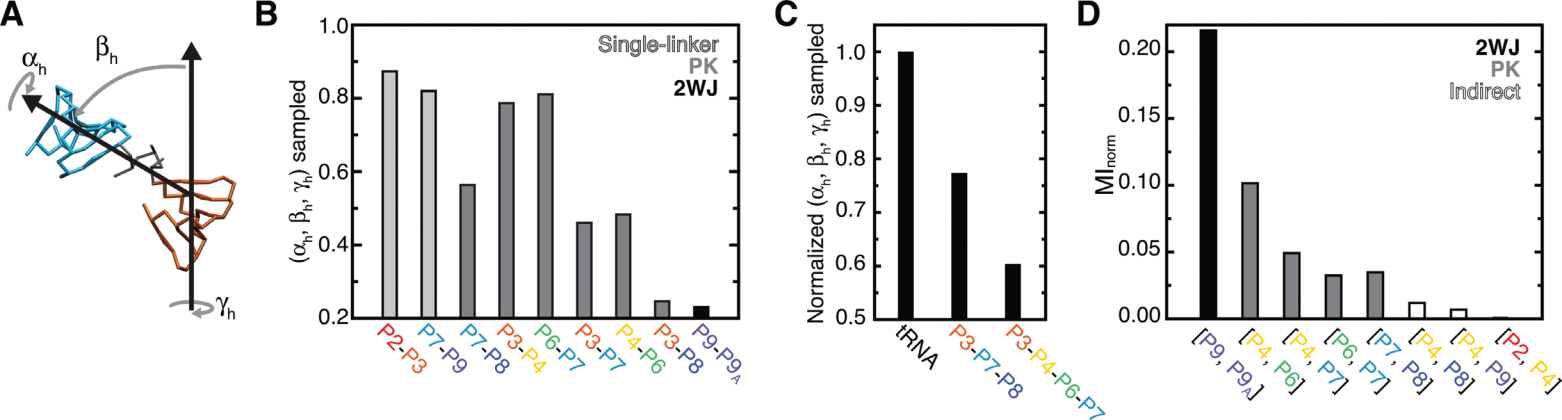
Quantification of topological constraints. (**A**) The (*α*_h_, *β*_h_, *γ*_h_) convention used to measure inter-helical conformation, illustrated using P3 and P7 from an example simulation snapshot. (**B**) The fraction of (*α*_h_, *β*_h_, *γ*_h_) space sampled between pairs of *Azoarcus* ribozyme helices. Light gray, dark gray and black bars are used to indicate helix pairs that are connected via a single-linker, pseudoknot, or standard two-way junction topology, respectively. Pseudoknot-linked pairs are ordered by the lengths of their connecting linkers. (**C**) The fraction of total 2 × (*α*_h_, *β*_h_, *γ*_h_) and 3 × (*α*_h_, *β*_h_, *γ*_h_) interhelical conformations sampled by the three-way and four-way pseudoknotted junctions normalized relative to tRNA (see Methods; (25)). (**D**) The MI_norm_ between the (*α*_h_, *β*_h_, *γ*_h_) distributions of different helices computed relative to P3; a complete set of MI_norm_ values is shown in Supplementary Figure S1. Helices that are linked via a standard two-way junction, pseudoknot or indirectly linked are indicated by black, dark gray and open bars.

To better characterize the effects of topological constraints on *Azoarcus* ribozyme conformation, we used Euler angles (*α*_h_, *β*_h_, *γ*_h_) to quantify the interhelical orientations sampled between pairs of helices across junctions (21,41). The angles *α*_h_ and *γ*_h_ specify the twists of the two helices, and *β*_h_ the inter-helical bend (Figure 2A). When discritized, these angles provide a finite coordinate space describing all possible interhelical conformations. We previously used this analysis to show that topological constraints limit individual two-way and four-way junctions to 7–27% and 3–9% of their theoretical conformational space (24–26). Notably, in the *Azoarcus* ribozyme, the three-way and four-way pseudoknotted junctions defined by P3, P4, P6, P7 and P8 comprise secondary structure motifs whose constraints have never been quantified.

Overall, Euler angle analysis indicates that pairs of helices in the *Azoarcus* ribozyme experience similar topological constraints as observed in tRNA (25). Due to relaxed steric constraints, helices linked by intervening single stranded regions can access a greater fraction of the (*α*_h_, *β*_h_, *γ*_h_) space (Figure 2B). For example, P2 and P9, which are only linked by 1–3 single-stranded nucleotides to the core of the molecule, are largely unconstrained and sample 82–87% of possible interhelical orientations relative to their nearest helix. Similarly, helices within the central four-way junction pseudoknot sample ∼80% of possible orientations when linked by intervening single strands, but 48% when directly linked (e.g. P4-P6 versus P6-P7 in Figure 2B). Strikingly, however, helices in the three-way pseudoknot are much more constrained. P3 and P7, and P7 and P8, only sample 45–56% of their possible relative orientations despite being linked by three and six single-stranded nucleotides, respectively. Moreover, P3 and P8 only sample 25% their possible relative orientations, indicating these helices experience topological constraints comparable in magnitude to those posed by a 3-nucleotide bulge, such as links P9 and P9A (Figure 2B). This high degree of topological restriction most likely arises from strong steric constraints that limit the conformational freedom of helices and linkers in pseudoknotted topologies.

In addition to limiting the conformation of individual pairs of helices, topological constraints greatly restrict the higher-order conformational space of pseudoknots. Computing the fraction of unique 2 × (*α*_h_, *β*_h_, *γ*_h_) combinations sampled between P3 and P7, and P3 and P8 reveals that only 4% of possible P3–P7–P8 pseudoknot conformations are accessible. Similarly, the P3–P4–P6–P7 pseudoknot samples only 5% of possible 3 × (*α*_h_, *β*_h_, *γ*_h_) conformations. Compared to the tRNA four-way junction (25), the P3–P7–P8 and P3–P4–P6–P7 pseudoknots are 23% and 40% more constrained, respectively, despite containing longer single-stranded loops (Figure 2C). Thus, as noted above, pseudoknots place uniquely strong topological constraints on RNA 3D conformation.

Further evidence that topological constraints globally restrict the ribozyme conformational space comes from analysis of the correlations between distinct sets of (*α*_h_, *β*_h_, *γ*_h_) angles. Normalized mutual information (MI_norm_) provides a general measure of correlation ranging from 0 (no correlation) to 1 (complete correlation). Consistent with the analysis above, computing MI_norm_ between the (*α*_h_, *β*_h_, *γ*_h_) conformations of pseudoknot-linked helices reveals small but significant correlations (0.03 < MI_norm_ < 0.11; Figure 2B). Surprisingly, residual correlations are also observed between P4 and P8, and P4 and P9 (MI_norm_ ≈ 0.01), which are separated by 1–2 intervening helices. By contrast, the conformation of the peripheral P2 helix is completely uncorrelated (MI_norm_ = 0) from other helices. Collectively, these analyses indicate that topological constraints act over long distances to limit the unfolded conformational space of the *Azoarcus* ribozyme.

### Topological constraints penalize formation of non-native tertiary contacts

In our previous studies of tRNA (25), we found that topological constraints can contribute to tertiary folding specificity by imposing large free energy penalties on forming non-native tertiary contacts. We thus explored whether topological constraints play a similar role in *Azoarcus* ribozyme folding. In particular, such a mechanism could help explain the specificity of ribozyme folding despite reliance on non-specific A-minor and base-triple motifs (12,13), and identical TL/TLR motifs between L2 and J8, and L9 and J5 (Figure 1A).

**Figure 3.**
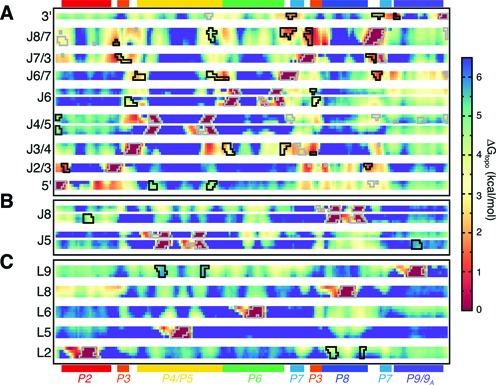
The Δ*G*_topo_ of forming different pairwise tertiary contacts in the *Azoarcus* ribozyme. All residues are shown along the *x*-axis, with selected single-stranded regions grouped along the *y*-axis according to whether they (**A**) participate in A-minor and base-triple interactions, (**B**) are tetraloop-receptors or (**C**) are tetraloops. Regions outlined in black correspond to bona fide long-range tertiary interactions. Regions that are proximal in the native structure but do not interact are outlined in gray.

We computed the free energy cost, Δ*G*_topo_, that topological constraints impose on forming different tertiary contacts from the probability that residues come within a feasible pairing distance (Equation 1). These calculations underestimate the true cost posed by topological constraints on forming a given interaction because they ignore interaction geometry. Nonetheless, the relative magnitudes of the derived Δ*G*_topo_ values provide valuable insight into the tertiary folding energy landscape. Consistent with our findings in tRNA (25), the penalty for forming different long-range contacts in the *Azoarcus* ribozyme is highly variable, with Δ*G*_topo_ varying from 0.5 kcal/mol to 6.5 kcal/mol (Figure 3). Strikingly, these penalties are generally greater for non-native contacts than native contacts (native contacts are outlined in black in Figure 3), indicating that topological constraints provide an inherent source of tertiary folding specificity. General features of the two classes of tertiary interactions—A-minor and base-triple interactions, and TL/TLR motifs—are discussed below.

Native A-minor and base-triple interactions form with the lowest Δ*G*_topo_ penalties, with Δ*G*_topo_ <3 kcal/mol and typically <2 kcal/mol (Figure 3A). Moreover, non-native contacts have large Δ*G*_topo_ penalties, generally >4 kcal/mol. As A-minor and base-triple interactions consist of only several hydrogen bonds and contribute at most −2 kcal/mol in stabilizing energy (45), the ∼2 kcal/mol ΔG difference encoded by topological constraints is sufficient to strongly disfavor formation of non-native contacts. Interestingly, native triple helix (TH) helix interactions involving J3/4 and P6, and J6/7 and P4, have particularly low Δ*G*_topo_ penalties, although these interactions also compete with local J3/4 to P4 and J6/7 to P6 contacts. The only native A-minor interaction with a large Δ*G*_topo_ penalty occurs between J4/5 and the 5′-loop; this is consistent with this interaction only forming subsequent to pairing between the 5′-loop and the 5′-exon (46,47), which places additional constraints on the 5′-loop that likely promote folding.

In contrast to A-minor/triple motifs, TL/TLR interactions possess Δ*G*_topo_ >3.5 kcal/mol and are endowed with less specificity (Figure 3B and C). For example, L2 forms non-native contacts with P7, P4, and P5 with Δ*G*_topo_ comparable to its native J8 receptor. Likewise, L9 forms non-native contacts with P6 and P7 with Δ*G*_topo_ < 3.5 kcal/mol, less than for its native interaction with J5 (Figure 3C). Apical loops that do not form interactions in the native structure also have the potential to form non-native contacts; the A-rich L8 loop forms contacts with J5 with Δ*G*_topo_ ≈ 4 kcal/mol, less than the Δ*G*_topo_ of the native L9/J5 interaction. Taken together, these results support that sequence plays a crucial role in the specificity of TL/TLR interactions (14,48).

**Figure 4.**
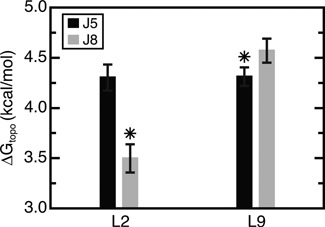
The Δ*G*_topo_ for forming different TL/TLR combinations. The native L9/J5 and L2/J8 interactions are indicated by asterisks. Values represent the mean and standard deviation of three simulations (the primary and two SI simulations).

Significantly, however, topological constraints do penalize formation of non-native TL/TLR pairs (Figure 4). Native L2/J8 contacts are favored by −0.8 kcal/mol over non-native L2/J5 interactions. From the perspective of J8, the native L2/J8 interaction is favored by −1.1 kcal/mol over the competing L9/J8 interaction. This native-specificity is not a trivial function of sequence proximity—mapped onto the linear chain, both L2 and L9 are ∼70 nucleotides closer to their non-native TLRs. The selectivity of the L2/J8 interaction can be partially rationalized by the fewer number of flexible pivots separating L2 and J8 along the secondary structure compared to L9 and J8 (two vs. four flexible pivots; Supplementary Figure S2). By analogy to a freely jointed chain model, this smaller number of pivots lowers the Δ*G*_topo_ for L2/J8 by -0.5 kcal/mol (Supplementary Information). The remainder of the ∼1.0 kcal/mol free energy gap between L2/J8 and L9/J8 interactions, as well as the selectivity of J5 for L9, arises from sterics and other asymmetric properties of the secondary structure like the varied lengths of helices. Thus, for these two TL/TLR motifs that have identical sequences, topological constraints provide a notable source of folding specificity.

We further explored whether the native-specificity of individual tertiary contacts translates into a preference for forming native versus non-native well-packed folds. In particular, we previously found that topological constraints prevent tRNA from forming multiple sets of non-native tertiary contacts, uniquely encoding the native state as the best-packed fold (25). Unfortunately, the failure of our simulations to sample compact states (Figure 1D) precluded such an analysis. In an attempt to increase sampling of compact conformations, we performed an additional simulation restraining the radius of gyration of the ribozyme to 32 Å. However, this restraint strongly disfavored P4/P6 stacking, which requires an end-to-end distance across P4/P6 of ∼100 Å. Given that P4/P6 stacking is critical for native folding, this *R*_g_-restrained simulation proved similarly inappropriate for testing whether topological constraints favor native compact folds.

### Large Δ*G*_topo_ penalties disfavor isolated TL/TLR folding

**Figure 5.**
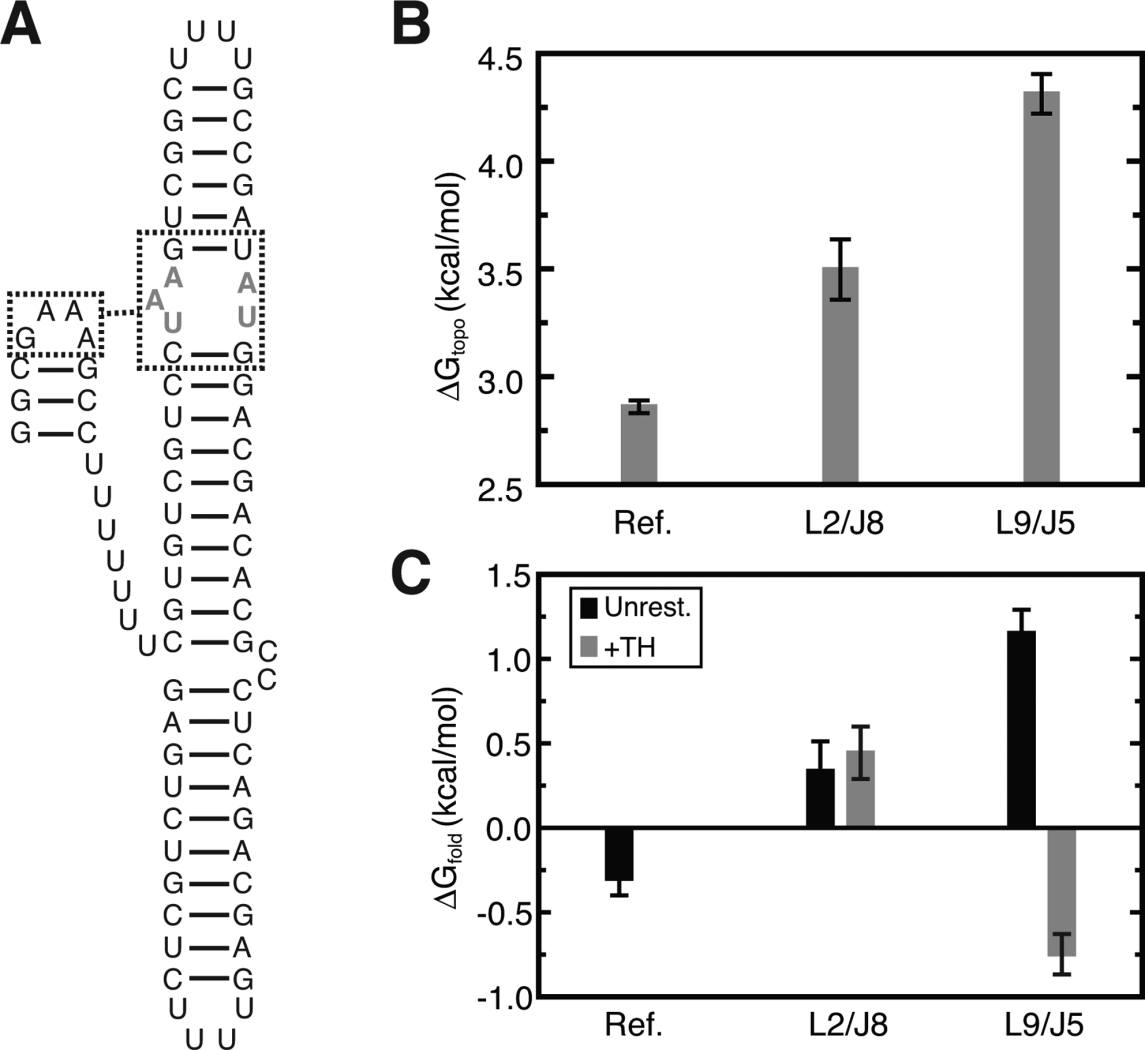
Comparison between the Δ*G*_topo_ for an isolated TL/TLR interaction and *Azoarcus* TL/TLR interactions. (**A**) Secondary structure of the simulated reference system (49). TLR residues that were restrained to the native receptor conformation are drawn in gray lettering. (**B**) Δ*G*_topo_ for the reference system and L9/J5 and L2/J8 interactions. Values for the reference represent the mean and standard deviation of three replicates. (**C**) Δ*G*_fold_ for the reference measured experimentally at 37°C and 1 mM Mg^2+^ (Figure 3 in (49)), and calculated for L9/J5 and L2/J8 via Equation (3) from unrestrained simulations (black). ΔG_fold_ of the TL/TLRs conditional on TH folding, estimated from TH-restrained simulations, is shown in gray bars.

Combined, our results indicate that topological constraints place strong entropic penalties on *Azoarcus* ribozyme tertiary folding, particularly for TL/TLR interactions. While TL/TLRs constitute the most stable RNA tertiary interaction motif (48), their stability in the context of large RNA molecules is less understood. We thus compared the Δ*G*_topo_ of the ribozyme TL/TLRs to a well-characterized reference system consisting of a TL linked by an oligonucleotide U tether to a TLR (Figure 5A) (48,49). TOPRNA simulations of the reference TL/TLR yielded Δ*G*^ref^_topo_ = 2.9 kcal/mol (note, as above, that this is an underestimate of the true value (50)). Thus, compared to the Δ*G*_topo_ = 3.5–4.3 kcal/mol for L2/J8 and L9/J5, the architecture of the *Azoarcus* ribozyme destabilizes TL/TLR motifs by 0.6–1.4 kcal/mol (Figure 5B).

This comparative analysis can be further extended to estimate the overall ΔG_fold_ of each ribozyme TL/TLR. Separating Δ*G*_fold_ into two components

(2) \begin{equation*} \Delta G_{{\rm fold}} = \Delta G_{{\rm other}} + \Delta G_{{\rm topo}} , \end{equation*}

where Δ*G*_other_ is a combination of all other free energy terms such as the favorable enthalpy of folding, and assuming Δ*G*_other_ is constant for TL/TLRs in different molecular architectures, the Δ*G*_fold_ of an arbitrary TL/TLR can be estimated through

(3) \begin{equation*} \Delta G_{{\rm fold}} = \Delta G_{{\rm fold}}^{{\rm ref}} - \Delta G_{{\rm topo}}^{{\rm ref}} + \Delta G_{{\rm topo}} \end{equation*}

Using the experimental value for Δ*G*^ref^_fold_ = −0.3 kcal/mol (49), this analysis predicts that Δ*G*_fold_(L2/J8) = 0.3 kcal/mol and Δ*G*_fold_(L9/J5) = 1.2 kcal/mol (Figure 5C).

Significantly, these predictions agree with the experimentally observed folding behavior of the *Azoarcus* ribozyme (20,51). L2/J8 folds early and independently from the rest of the ribozyme, consistent with the prediction that Δ*G*_fold_ ≈ 0 for independent L2/J8 folding. By contrast, L9/J5 folding is tightly coupled to global folding of the ribozyme, which is consistent with our prediction that isolated L9/J5 folding is substantially disfavored. The comparative instability of L9/J5 is also supported by the conservation of high affinity GAAA/11-nt-receptor sequences at L9/J5, whereas lower affinity TL/TLR sequences are often substituted at L2 and J8 (14,30,52). Together, this analysis supports that Δ*G*_topo_ is a key component of the overall folding free energy of the *Azoarcus* ribozyme.

### Reductions in Δ*G*_topo_ upon tertiary contact formation cooperatively promote native folding

The observation that large Δ*G*_topo_ penalizes individual folding of TL/TLR motifs suggests that a cooperative folding mechanism is required to stabilize the native fold. Indeed, recent experiments have demonstrated strong thermodynamic couplings between tertiary interactions play a critical role in stabilizing the *Azoarcus* ribozyme (27). We therefore explored whether folding of individual tertiary contacts promotes folding of additional contacts by reducing Δ*G*_topo_.

A wealth of experiments has shown that the core triple helix (TH) motif, comprising a series of base triples between J6/7 and P4, and J3/4 and P6, is essential for global ribozyme folding (27,53–55). Our simulations indicate that TH interactions have low Δ*G*_topo_ penalties and are thus likely to form early along the equilibrium folding pathway (Figure 3). To measure cooperative stabilization effected by TH folding, we computed

**Figure 6.**
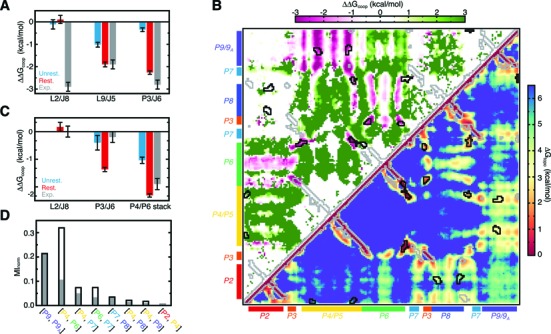
Topological constraints give rise to cooperativity between distal tertiary interactions. (**A**) Cooperativity between the TH and indicated tertiary interactions compared to values measured experimentally (27). Errors represent the standard deviation over the unrestrained main text and two SI simulations, and three replicate restrained simulations. (**B**) The Δ*G*_topo_ of forming pairwise tertiary contacts in the TH-restrained *Azoarcus* ribozyme (lower triangle), and the ΔΔ*G*_coop_ computed relative to the unrestrained simulation (upper triangle). Regions outlined in black correspond to bona fide long-range tertiary interactions. Regions that are proximal in the native structure but do not interact are outlined in gray. (**C**) Cooperativity between L9/J5 and the indicated tertiary interactions, compared to values measured experimentally (27). A lack of sampling precluded computation of ΔΔ*G*_coop_ between L9/J5 and L2/J8 from the unrestrained simulations. (**D**) The MI_norm_ between the (*α*_h_, *β*_h_, *γ*_h_) distributions of selected helices computed relative to P3 for the TH-restrained (open black bars) and unrestrained (filled gray bars) simulations. To normalize for differences in simulation length, MI_norm_ values for the unrestrained simulation were computed using only the first 10^9^ simulation steps.

(4) \begin{equation*} \Delta \Delta G_{coop} (x,TH) = \Delta G_{topo} (x|TH) - \Delta G_{topo} (x), \end{equation*}

where ΔΔ*G*_coop_(*x*, TH) is the cooperativity between a tertiary contact *x* and the TH, and Δ*G*_topo_(*x* | TH) is the free energy of forming *x* conditional on TH folding. Δ*G*_topo_(*x* | TH) was computed both from our original unrestrained simulation, using P4/P6 stacking as a proxy of TH formation, and also more precisely from a new simulation where the TH was restrained to its native conformation. Both calculations indicated strong cooperativity between the TH and other native interactions, strengthening by −1 to −2 kcal/mol when the full TH is restrained to its native geometry (Figure 6A). Strikingly, ΔΔ*G*_coop_(L9/J5, TH) = −1.9 kcal/mol from the TH-restrained simulation, identical to the value measured experimentally (Figure 6A). The ΔΔ*G*_coop_ = −2.3 kcal/mol between the TH and J3/P6 A-minor interaction is also in good agreement with experimental measurements (Figure 6A). However, we fail to observe cooperativity between L2/J8 and the TH, which likely originates from the interdependent network of tertiary interactions made between the TH, the 5′-loop, J2/3, J8/7 and P2 not captured in our simulation.

Equally significant, TH folding also strongly antagonizes non-native contacts by 1–2 kcal/mol, greatly increasing the specificity of the folding landscape (Figure 6B). Of particular note, non-native L9/J2 TL/TLR interactions are penalized by ∼1 kcal/mol. Combined with the cooperative stabilization of the native L9/J5 interaction, this results in a >3 kcal/mol energy gap between native and non-native L9 TL/TLR contacts, compared to the 0.3 kcal/mol gap observed in the absence of the TH (Figure 4).

The observed cooperativity derives from the ability of topological constraints to propagate local constraints posed by TH folding into global changes in the ribozyme conformational landscape. Euler angle analysis of the TH-restrained simulation revealed 1.1× to 5× decreases in the fraction of conformations sampled by individual pairs of helices and by both pseudoknots (Supplementary Figure S3). Mutual information analysis also revealed that the TH strengthens the long-range correlations between helices by as much as 2-fold (Figure 6D, Supplementary Figure S3).

As a test of the generality of this topological constraint induced cooperativity, we performed similar analysis of the effects of L9/J5 formation on the folding landscape. Calculation of ΔΔ*G*_coop_ from Equation (4) using both unrestrained and L9/J5-restrained simulations revealed stabilization of other tertiary interactions by 0 to −2.3 kcal/mol (Figure 6C). As observed for the TH, cooperativities increased substantially in the L9/J5-restrained simulation, reflecting the importance of precise L9 positioning in stabilizing the native fold. Consistent with experiment, no cooperativity was observed between L2/J8 and L9/J5. Interestingly, we observe −1.4 kcal/mol between P3/J6 and L9/J5 in the restrained simulation, significantly more than the −0.2 kcal/mol measured experimentally (27). Also similar to the TH, L9/J5 folding strongly antagonizes non-native interactions by 1–3 kcal/mol (Supplementary Figure S4).

Taken together, our analysis shows that additional constraints posed by individual tertiary interactions cooperatively increase the specificity and stability of the tertiary free energy landscape. As noted previously (20,27,28), the −1 to −3 kcal/mol energetic couplings between tertiary interactions are comparable or greater than the Δ*G*_fold_ contributed by individual contacts. Supporting the criticality of these couplings to the stability of the native fold, we predict from Equation (3) that Δ*G*_fold_(L9/J5 | TH) = −0.7 kcal/mol, whereas Δ*G*_fold_(L9/J5) = 1.2 kcal/mol in the absence of the TH (Figure 5C). Thus, as was observed for tRNA (25,26), topological constraints play a critical role in both the specificity and stability of the *Azoarcus* ribozyme.

## DISCUSSION

The mechanisms underlying RNA tertiary folding specificity and cooperativity have long been poorly understood. In particular, tertiary interactions are relatively non-specific, raising the key question of how RNAs avoid tertiary misfolding. Our simulations demonstrate that topological constraints imposed by native secondary structure provide a powerful source of folding specificity in the *Azoarcus* ribozyme. By significantly constraining the accessible 3D conformational space, topological constraints encode large, variable Δ*G*_topo_ penalties that discriminate against formation of non-native tertiary interactions. Additional constraints imposed by folding of the TH further increase the specificity of the folding landscape, cooperatively stabilizing native interactions and antagonizing non-native interactions. Thus, analogous to the funneled folding landscapes of proteins (56), our results argue that secondary structure helps encode a cooperative folding funnel in the *Azoarcus* ribozyme.

As might be expected given their lack of sequence-specificity (10–13), A-minor and base-triple tertiary interactions appear to be particularly reliant on topological constraints for specificity. Native A-minor and base-triple interactions occur between regions that form with low Δ*G*_topo_ (<3 kcal/mol), whereas non-native contacts are discriminated against via large Δ*G*_topo_ penalties (Figure 3). Interestingly, base-triple interactions in tRNA similarly have low ΔG_topo_ penalties and high specificity from topological constraints (25). We suggest that A-minor and base-triple-type interactions are largely ‘opportunistic’, occurring between loops and helices that are topologically local with few alternative interaction partners.

By contrast, TL/TLR interactions have both high Δ*G*_topo_ penalties and lower topological-constraint-encoded specificities. The high thermodynamic stability and unique sequence specificity of TL/TLR motifs is thus indispensable (14,48), and explains the selection pressure for GAAA/11-nt-receptor sequences at L2/J8 and L9/J5 (30,52). Our results also point to a role for negative design, as the non-GNRA sequences of loops L6 and L8 likely help negate the otherwise low Δ*G*_topo_ of forming non-native contacts with J2 and J5. Nevertheless, topological constraints do appear to play an important role in discriminating against non-native TL/TLR interactions (Figures 4 and 6). Given their significant enthalpies, formation of non-native TL/TLR interactions would pose large kinetic barriers to native tertiary folding (49,57). We thus surmise that topological constraints serve as a common source of specificity in large RNAs that contain multiple TL/TLR motifs.

Consistent with a model where formation of low Δ*G*_topo_ A-minor and base-triple interactions helps further funnel the folding landscape, TH folding increases both the specificity and stability of other tertiary interactions. Critically, TH folding stabilizes L9/J5 by −1.9 kcal/mol, matching the cooperativity measured between these motifs experimentally (27), and sufficient to shift the Δ*G*_fold_ of L9/J5 from +1.2 to −0.7 kcal/mol. Such a sequential ‘inside-out’ folding pathway likely offers several advantages. First, formation of A-minor/triple interactions helps offset the large entropic penalty associated with the first stages of compaction. Second, delayed folding of L9/J5 may help prevent formation of topologically frustrated intermediates. Notably, experimental studies have shown that delayed folding of TL/TLR motifs such as L9/J5 speeds acquisition of the final native fold by minimizing frustration (51). Prior non-equilibrium folding simulations of the *Azoarcus* ribozyme also observed a large fraction of frustrated species (43).

While a TH-initiated folding pathway is likely favored, our analysis of L9/J5 shows that progressive funneling of the folding landscape upon tertiary contact formation is likely path-independent. We note that since our restrained simulations begin from the native fold, we cannot rule out that the possibility that native L9/J5 (or TH) contacts also form in topologically frustrated species. This would antagonize rather than promote further folding in these species. However, the fact that our unrestrained simulations give similar measures of cooperative stabilization (Figure 6) argues that the primary effect of both L9/J5 and TH folding is to globally funnel the molecule towards the native fold. Such path independence is a classic attribute of folding funnels (58).

Within the context of the overall free energy landscape, Δ*G*_topo_ is likely most significant in shaping the initial collapse of the ribozyme to an ensemble of near-native intermediates. Δ*G*_topo_ penalties are insufficient to discriminate against the huge number of near-native conformations possessing local differences in hydrogen bonding and helical orientation. Complete native folding requires resolution of these differences, and is driven by specific Mg^2+^ ion binding and precise consolidation of native tertiary interactions (8,18). Indeed, the cooperativities measured from our simulations generally agree well with those measured for the intermediate ensemble (Figure 6), but differ substantially from those measured in the native fold (27). These comparisons are imprecise, because experimentally derived cooperativites represent the sum of all energy terms rather than just Δ*G*_topo_. Nevertheless, the consistency between our simulations and experiment support that topological constraints play a central role in stabilizing compact intermediates (27). Entropic/enthalpic tradeoffs made while maximizing tertiary interactions are in turn likely responsible for the changes in tertiary cooperativity observed in the native fold (27).

A limitation of our study, particularly with regards to analyses of folding pathways, is the assumption that secondary structure folding strictly precedes tertiary folding. Studies have indicated that secondary structure folding is at least partially coupled to tertiary structure folding in the *Azoarcus* ribozyme (20). In other RNAs, tertiary interactions can bias the order of helix assembly (59) and stabilize otherwise unstable secondary structures (19,60); exploring how topological constraints of partially folded or non-native secondary structures affect folding is an important topic for future study. Electrostatics and attractive interactions, which we ignore, also play critical roles in RNA tertiary folding (8,61,62). The heterogeneous folding kinetics of the *Azoarcus* ribozyme underscores that the energy landscape experienced by the real RNA is much more complex than modeled here (51,63–65). However, emphasizing the importance of secondary structure in shaping tertiary folding, secondary structure destabilizing mutations decrease the tertiary folding rate of the *Azoarcus* ribozyme by 1000-fold (44,64). Inversely, stabilizing secondary structure increases the folding rate in the related *Tetrahymena* ribozyme (66,67). Regardless, Δ*G*_topo_ constitutes a fundamental component of the free energy landscape and will contribute to the specificity of the native fold independent of the folding pathway.

The concept of minimal frustration—that evolution favors sequences that mutually support the native fold and discriminate against non-native folds—has served as a central organizing concept in protein folding (56,58). In particular, the importance of mutual ‘consistency’ between secondary structure and tertiary structure has long been recognized in proteins (68,69). It makes intuitive sense that similar principles should apply to RNA (3). Secondary structure interactions supply the overwhelming majority of energy available to stabilize an RNA fold. Combined with the low specificity of many RNA tertiary interactions, and the generally hierarchical nature of RNA folding, there should be particularly strong pressure to select secondary structures that favor tertiary folding. Our results help provide a rigorous free energy framework for understanding the overwhelming conservation of the group I intron secondary structure core (70), as well as Michel and Westhof's striking observation that this core largely encodes group I intron tertiary structure (30). We suspect that similar mutual consistencies between secondary and tertiary structure will prove to be widespread in the RNA world.

## Supplementary Material

SUPPLEMENTARY DATA
