# Supplementary material for: Secondary structure encodes a cooperative tertiary folding funnel in the Azoarcus ribozyme
Source: Nucleic Acids Res. 2015 Oct 19;44(1):402–12. doi: 10.1093/nar/gkv1055 (PMC4705646; doi:10.1093/nar/gkv1055)
Supplement: SUPPLEMENTARY DATA [file supp_44_1_402__index.html]

Secondary structure encodes a cooperative tertiary folding funnel in the Azoarcus ribozyme — SUPPLEMENTARY DATA 

# Secondary structure encodes a cooperative tertiary folding funnel in the *Azoarcus* ribozyme

## SUPPLEMENTARY DATA

- SUPPLEMENTARY DATA
