# Supplementary material for: Secondary structure encodes a cooperative tertiary folding funnel in the Azoarcus ribozyme
Source: Nucleic Acids Res. 2015 Oct 19;44(1):402–12. doi: 10.1093/nar/gkv1055 (PMC4705646; doi:10.1093/nar/gkv1055)
Supplement: SUPPLEMENTARY DATA [file supp_gkv1055_nar-02364-r-2015-File008.pdf]

SUPPLEMENTARY INFORMATION

Secondary structure encodes a cooperative tertiary folding funnel in the *Azoarcus* ribozyme

Anthony M. Mustoe[1,§], Hashim M. Al-Hashimi[2], Charles L. Brooks III[1,3,*]

Departments of [1]Biophysics and [3]Chemistry, University of Michigan, Ann Arbor, MI, 48109

[2]Department of Biochemistry and Chemistry, Duke University School of Medicine, Durham, NC, 27710

[§]Present address: Department of Chemistry, University of North Carolina, Chapel Hill, NC, 27599

*To whom correspondence should be addressed: brookscl@umich.edu

Simulations using alternative internal loop restraints

Two additional simulations we performed to explore the effects of alternative internal loop pairing. Each simulation used the same initial coordinates and simulation parameters as described in the main text, with internal loop restraints as described below:

i) To test the importance of exact geometry for native non-canonical pairs, we restrained the backbone dihedrals of internal loops to their crystal structure values (1). As an exception, J5 and J8 were restrained to the 'zipper' conformation adopted by 11-nt receptors in the absence of a docked GAAA tetraloop (PDB 1TLR (2)). Restraints were given force constants K=50 kcal/mol and periodicity of 1, leaving the backbone of bulged residues unrestrained. For J5 and J8, restraints were placed along all backbone dihedrals. Unlike all other simulations, "filler" beads were not placed between non-canonically paired nucleotides.

ii) To explore the consequences of using naïvely chosen and potentially non-native pairing restraints (as would be the case if modeling an RNA with unknown structure), we used MC-fold (3) to predict pairs for each internal loop of the *Azoarcus* ribozyme. The P2, P4/5, P6, P7, and P9 hairpin sequences were input to the MC-fold webserver using default settings. Non-canonical pairs were identified from the lowest energy base-paired structure of each hairpin that was consistent with the native secondary structure. This procedure yielded the following internal loop pairs: C11-A21, A48-A78, A49-A77, A50-C76, A54-U73, A55-A72, A88-A112, G90-A111, U92-U109, A140-U153, A141-A152, G173-A193, and A174-A189. These pairs were restrained using the same scheme as described in the main text.

For each simulation, we computed the fraction of (α_h, β_h, γ_h) angles sampled between helices, the mutual information between different (α_h, β_h, γ_h) angles, and the ΔG_{topo} of forming different tertiary interactions. All analyses revealed negligible differences when compared to the main text simulation (for example, see error bars in Figure 4, 6).

Free energy of forming end-to-end contacts for an ideal chain

For an ideal polymer chain, the probability of forming end-to-end contacts can be computed according to:

$$P_{ee} = \left[\frac{3}{2\pi N b^2}\right]^{3/2} \int_0^d \exp\left(\frac{-3r^2}{2Nb^2}\right) 4\pi r^2 dr \qquad \textbf{[S1]}$$

where N is the number of monomers, b is the monomer length, and d is the end-to-end contact distance cutoff. When $d = b$ and $1/N << 1$, [S1] reduces to

$$P_{ee} \approx \left(\frac{6}{\pi}\right)^{1/2} N^{-3/2}, \qquad \textbf{[S2]}$$

with P_{ee} only depending on N (4). The free energy cost of making end-to-end contacts, ΔG_{ee}, can then be obtained through equation **[1]** in the main text.

L2 and J8 are separated by two pivot points in the secondary structure, and hence can be roughly interpreted as the termini of a 3-mer, where each monomer is either a rigid secondary structure helix, or a base pair across a helix (Figure S2). Similarly, L9 and J5 can be interpreted as the termini of a 5-mer. Using the approximate P_{ee} solution, **[S2]**, the $\Delta\Delta G_{ee}$ between a 5-mer and 3-mer is ~0.6 kcal/mol. Because N is small, and $d \neq b$, we also computed the $\Delta\Delta G_{ee}$ using P_{ee} values obtained from numeric integration of **[S1]**. Setting d=14 Å (the distance across a base pair in TOPRNA) and b=15 Å (the average across all helix lengths between L2/J8 and L9/J5), we obtain $\Delta\Delta G_{ee} \approx 0.5$ kcal/mol. Using different exponents for N in [S2] to account for excluded volume effects minimally affects the calculated $\Delta\Delta G_{ee}$.

Supplementary References

1. Adams, P.L., Stahley, M.R., Kosek, A.B., Wang, J. and Strobel, S.A. (2004) Crystal structure of a self-splicing group I intron with both exons. *Nature*, **430**, 45-50.
2. Butcher, S.E., Dieckmann, T. and Feigon, J. (1997) Solution structure of a GAAA tetraloop receptor RNA. *Embo J*, **16**, 7490-7499.
3. Parisien, M. and Major, F. (2008) The MC-Fold and MC-Sym pipeline infers RNA structure from sequence data. *Nature*, **452**, 51-55.
4. Dill, K.A. and Bromberg, S. (2003) *Molecular driving forces: statistical thermodynamics in chemistry and biology*. Garland Science.
5. Mustoe, A.M., Al-Hashimi, H.M. and Brooks, C.L., III. (2014) Coarse grained models reveal essential contributions of topological constraints to the conformational free energy of RNA bulges. *J Phys Chem B*, **118**, 2615-2627.

Supplementary Figures

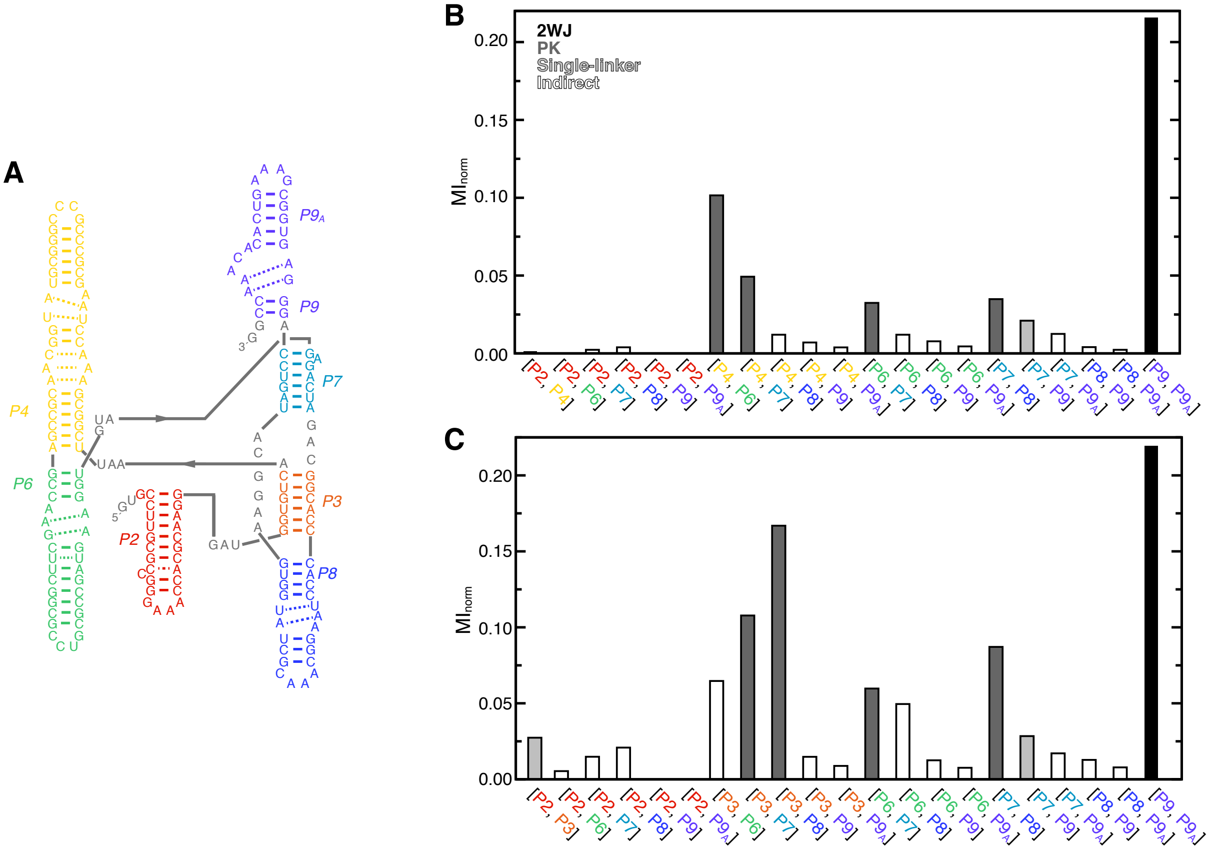

Figure S1: The MI_{norm} between all helices in the unfolded *Azoarcus* ribozyme. (**A**) Secondary structure cartoon showing helix labeling. (**B**) The (α_h, β_h, γ_h) distributions of different helices computed relative to P3. (**C**) The MI_{norm} between the (α_h, β_h, γ_h) distributions of different helices computed relative to P4. Black, dark gray, light gray, and white bars are used to denote helices that are linked by standard two-way junctions, pseudoknots, single-linkers, and indirectly, respectively. Comparisons between (**B**) and (**C**) show that use of P4 as the internal reference helix yields increased MI_{norm} values, particularly between P7 and P8. This increase reflects coupled large amplitude motions of P3, P7, P8, and P9 relative to P4 that are removed when P3 is used as the internal reference.

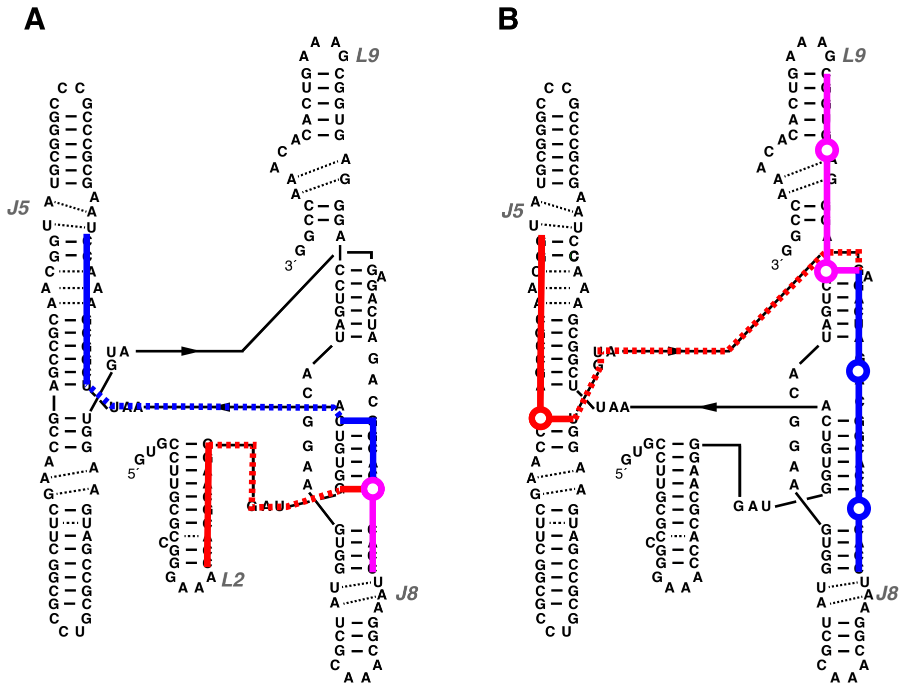

Figure S2: Diagram of the secondary structure pivots separating **(A)** L2 and **(B)** L9 from potential TLRs. The shortest secondary structure path to the native TLR is traced in red, and the shortest path to the non-native TLR is traced in blue; portions where the two paths overlap are colored magenta. Circles denote pivots, and dashed lines denote extended single strands, which are also counted as a single pivot. Note that 1-nt bulges were not counted as pivots because their conformational freedom is severely limited by topological constraints (5). In (B), the non-native path between L9 and J8 could alternatively be drawn through J8/7, yielding only three pivots. However, the tight coupling between P3 and P8 and long length of J8/7 makes the alternative path through P3 better for approximating the degrees of freedom separating L9 and J8.

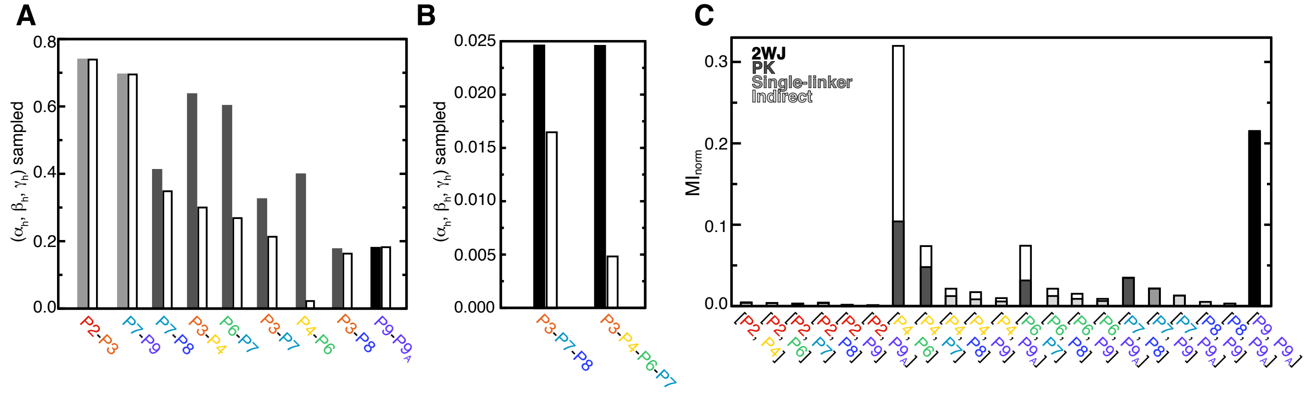

Figure S3: Euler angle analysis of the TH-restrained simulation (open bars) compared to the unrestrained simulation (filled bars). (**A**) Fraction of inter-helical angles sampled between pairs of helices connected via a single-linker (medium gray), pseudoknot (dark gray), or standard two-way junction topology (black). (**B**) Fraction of Euler space sampled by the three- and four-way pseudoknots. (**C**) The MI_{norm} between the (α_h, β_h, γ_h) distributions of different helices computed relative to P3. Helices connected via a two-way junction, pseudoknot, single linker, or indirectly are denoted using black, dark gray, medium gray, and light gray, respectively. We note that MI_{norm} computed using P4 as the internal reference helix (as in Figure S1C) either shows no increase, or decreases modestly in the TH-restrained simulation; this is because the MI_{norm} from the vantage of P4 is dominated by global motions of P3, P7, P8, and P9, which are attenuated by the additional constraints posed by the TH. Values shown for the unrestrained simulation in (A-C) were computed using only the first 10^9 simulation steps and hence are decreased relative to those reported in the main text; this undersampling is needed to perform fair comparisons to the shorter TH-restrained simulation.

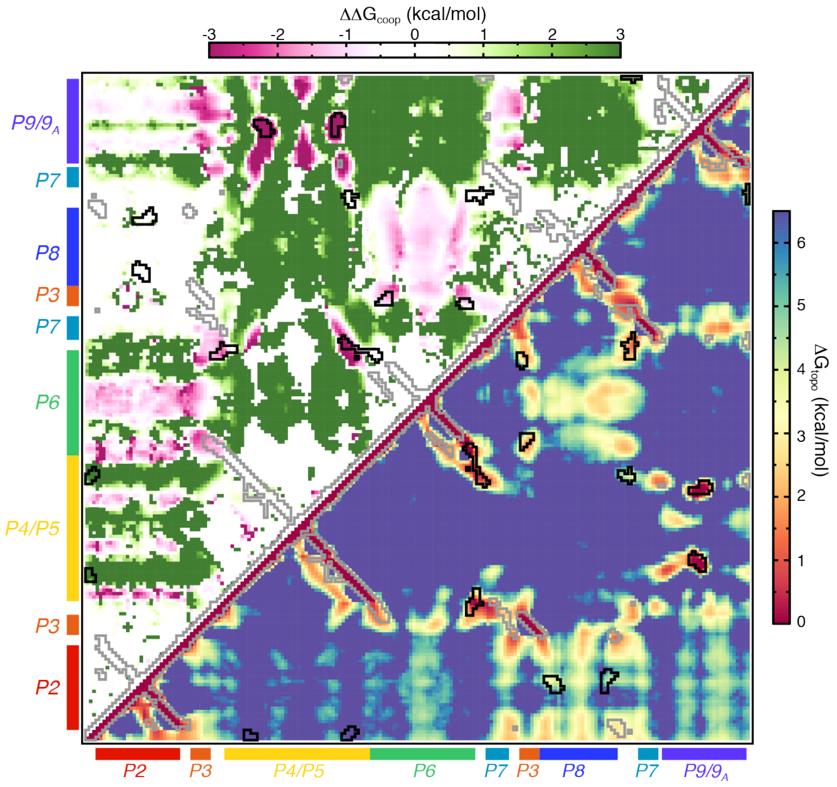

Figure S4: The ΔG_{topo} of forming pairwise tertiary contacts in the L9-restrained *Azoarcus* ribozyme (lower triangle), and the $\Delta\Delta G_{coop}$ computed relative to the unrestrained simulation (upper triangle). Regions outlined in black correspond to bona fide long-range tertiary interactions. Regions that are proximal in the native structure but do not form true tertiary interactions are outlined in gray.
